# Mirror, Mirror by the Stairs: The Impact of Mirror Exposure on Stair versus Elevator Use in College Students

**DOI:** 10.3389/fpubh.2016.00080

**Published:** 2016-04-25

**Authors:** Katie L. Hodgin, Dan J. Graham

**Affiliations:** ^1^Colorado State University, Fort Collins, CO, USA

**Keywords:** stairs, elevators, physical activity, mirrors, self-awareness

## Abstract

Previous research has indicated that self-awareness-inducing mirrors can successfully incite behaviors that align with one’s personal values, such as helping others. Other research has found a large discrepancy between the high percentage of young adults who report valuing the healthfulness of physical activity (PA) and the low percentage who actually meet PA participation standards. However, few studies have examined how mirror exposure and both perceived and actual body size influence highly valued PA participation among college students. The present study assessed stair versus elevator use on a western college campus and hypothesized that mirror exposure would increase the more personally healthy transportation method of stair use. In accordance with previous research, it was also hypothesized that males and those with a lower body mass index (BMI) would be more likely to take the stairs, and that body size distorting mirrors would impact the stair–elevator decision. One hundred sixty-seven students (51% male) enrolled in an introductory psychology course were recruited to take a survey about their “transportation choices” at an indoor campus parking garage. Participants were individually exposed to either no mirror, a standard full-length mirror, or a full-length mirror manipulated to make the reflected body size appear either slightly thinner or slightly wider than normal before being asked to go to the fourth floor of the garage for a survey. Participants’ choice of floor-climbing method (stairs or elevator) was recorded, and they were administered an Internet-based survey assessing demographic information, BMI, self-awareness, perceived body size, and other variables likely to be associated with stair use. Results from logistic regression analyses revealed that participants who were not exposed to a mirror [odds ratios (OR) = 0.37, 95% CI: 0.14–0.96], males (OR = 0.33, 95% CI: 0.13–0.85), those with lower BMI (OR = 0.84, 95% CI: 0.71–0.99), those with higher exercise participation (OR = 1.09, 95% CI: 1.02–1.18), and those engaging in more unhealthy weight-control behaviors (OR = 1.55, 95% CI: 1.14–2.11) showed increased odds of taking the stairs. Implications and future directions are discussed.

## Introduction

The span and significance of health benefits resulting from participation in physical activity (PA) are well documented. Along with aiding in the prevention of obesity and weight-related diseases (1), regular PA has been linked to improvements in cardiorespiratory fitness, blood pressure, flexibility, strength, and psychological functioning (2). Despite such health benefits and research revealing that a majority of young adults aged 18–25 years consider regular exercise to be healthy (3), recent national data suggest that only 20.3% of adults aged 18 years and older met the Physical Activity Guidelines for both aerobic and muscle-strengthening PA in 2012 (4). It is clear from these data that young adults value the healthfulness of, and may even intend to engage in PA (3), but are not actually behaving consistently with their attitudes.

Research has identified the age period of 18–29 years as a time in which adults are especially vulnerable to unhealthy weight gain and significant declines in PA (5). It is during this key period of young adulthood that approximately 68% of high school graduates attend college (6) and experience significant lifestyle changes that may substantially impact their health. For example, <35% of college students meet PA recommendations (7). Many researchers have recognized the first (freshman) year of college as a “critical period” for weight gain and associated unhealthy dietary and activity behaviors (8–11). Specifically, college students gain 3.75 pounds during their freshman year (8, 10) and perform an average of approximately 193 fewer minutes of vigorous PA per week than they performed in high school (12). Interestingly, one study found that caloric intake significantly decreased over the course of participants’ first semester at college, suggesting that a decline in PA is at least partly responsible for weight and fat gain in this population (9).

### Stair Use

One approach that has been used to promote routine PA is encouraging stair use in multilevel environments (versus using an elevator or escalator). Stair promotion has been attempted in various public settings, including subway stations (13), shopping malls (14, 15), and office buildings (16, 17). Laboratory research on caloric expenditure has found that climbing stairs requires 8.6 times more energy expenditure than the resting state (18). Furthermore, stair climbing meets the American College of Sports Medicine’s minimum PA intensity requirements for health gains due to the cardiovascular responses that result (19). Clearly, the exploration of factors associated with stair use versus less-active elevator or escalator use has great potential for informing public health researchers and helping individuals increase their energy expenditure in a convenient and inexpensive way. College campuses may be an ideal setting for researching factors associated with stair use in an effort to increase leisure-time PA among students because of their many residence and academic buildings with multiple floors.

Along with exploring demographic characteristics related to one’s stair use, factors that can actually be modified, such as the built environment, should be considered. Although some research has successfully increased stair use by improving the aesthetic feel of stairwells [e.g., Ref. (16)] and including point-of-decision signs to encourage stair over escalator use in public locations [e.g., Ref. (13)], the effects of such built environment modifications may not be long-lasting as their novelty wears off. A better approach may be to add something simple to the built environment that has been shown to motivate people to engage in certain behaviors that they personally value, such as using the more active transportation method of stairs when ascending floors. Using a mirror to serve as both an aesthetic feature and point-of-decision prompt in the built environment where people have a choice between floor-climbing methods may be an easier, more sustainable way to subtly encourage stair use. Mirrors may induce behavior change in a way that is more consistent with one’s attitudes and values by directing attention toward the self and increasing one’s awareness of personal values (20).

### Self-Awareness

Objective self-awareness (OSA) theory (21) operates under the notion that when attention is directed toward the self, OSA is induced and a subsequent comparison of the self to a “standard” is made. Duval and Wicklund (21) define “standard” as the combination of what the individual considers to be the correct behavior, attitudes, and traits of a person. If there is a discrepancy between the self and the standard after comparison, the individual will experience unpleasant feelings (i.e., cognitive dissonance) and will therefore try to close the perceived gap. As a result of this drive for consistency between the self and the standard, self-awareness has been found to be a moderator of the relationship between attitudes and behavior (22, 23) such that attitudes are more predictive of behavior when the individual is aware of the self. For example, the presence of a mirror has been shown to increase individuation and to facilitate behavior that has been made more salient and is seen as a standard of correct behavior (24). Although the use of mirrors to induce self-awareness has been found to successfully promote positive behavior change, few studies have distinguished between different categories of self-awareness and investigated the types of mirrors required to induce such categories.

It is important to note that research has demonstrated self-awareness as a separate state from self-consciousness. While self-consciousness is typically believed to be more of a stable, personality-like trait (25, 26), self-awareness depends on the situation and is considered highly changeable (26). Specifically, public self-awareness involves a focus on the aspects of the self that can be seen by other people, such as physical features, whereas private self-awareness involves more of a focus on internal aspects of the self, such as memories or feelings of pain (27). Public self-awareness, then, is most likely to lead to socially acceptable behavior change as a result of individuals attempting to relieve discomfort felt from possible social evaluation (28).

Govern and Marsch (20) note that different sizes of mirrors induce either public or private self-awareness. Because public self-awareness involves a heightened focus on aspects of the self that are observable by others, full-length mirrors that show the whole body, in contrast to small mirrors showing only the face, are used to induce this type of awareness. Although previous OSA research has studied the role of mirrors in producing helping behaviors, few studies have investigated the role of self-awareness through the use of mirrors in increasing physically active behaviors such as stair use.

### Body Size and Physical Activity

Research has identified important demographic characteristics that may encourage or deter physically active behaviors. In regards to one’s sex, Poobalan and colleagues (3) found that males tend to engage in more exercise in general, and Eves (29) observed that more males took the stairs than females. In addition, more normal-weight individuals were seen taking the stairs than overweight individuals (29). The body weight effect seen in such observational studies of stair use may be explained by Proffitt’s (30) “economy of action” theory, which postulates how people will visually perceive distances and slopes depending on their physical resources, with the goal to act in an energy-efficient manner (not to have energy expenditure exceed energy consumption). For example, Sugovic et al. (31) found that those with more body weight perceived distances to be farther.

Other, seemingly contradictory research on self-perception of body size among adolescents indicates that those who are and/or those who perceive themselves to be more overweight can be more likely to engage in weight-management behaviors but may do so using extreme or unhealthy approaches (32, 33). Thus, it could be that heavier individuals have a greater desire to lose weight, so they engage in more frequent or extreme dieting and exercise behaviors. To further explore this issue, the present study assesses participants’ healthy (e.g., exercising more, eating more fruits and vegetables) and unhealthy (e.g., fasting, taking laxatives) behaviors typically associated with weight control, and how each of these types of behaviors relates to college students’ stair use. Additionally, because the somewhat conflicting findings regarding the relationship between one’s perception of their body size and their PA behaviors make it reasonable to assume that seeing either a thinner-than-usual or wider-than-usual reflected body size will influence one’s decision to take the stairs instead of the elevator up several floors, the present study aims to discover how a body-thinning mirror influences stair use compared to a body-widening mirror.

In light of the previous research on PA, mirrors, and body size, the present study offers four hypotheses:

Hypothesis 1: a higher percentage of college students will use the stairs in conditions that expose them to a mirror than in a no-mirror condition.
Hypothesis 2: a higher percentage of males than females will use the stairs.
Hypothesis 3: body mass index (BMI) will be inversely associated with stair use, such that those with higher BMI will be less likely to use the stairs than those with lower BMI.
Hypothesis 4: body size-distorting mirrors will impact stair use.

## Materials and Methods

### Participants

One hundred seventy-four undergraduates (89 males, 85 females) enrolled in an introductory psychology course at a large public university took part in this study. The study occurred in a parking garage on campus and was advertised to students as “psychological influences on transportation choices.” Participants who completed the study were awarded research participation credit for the introductory psychology course in which they were enrolled. Institutional Review Board (IRB) approval was obtained prior to commencement of the study.

### Research Design

This study utilized a quasi-experimental design. Exposure to the presence of a mirror was manipulated to create four conditions: a standard mirror, a thinning mirror, a widening mirror, and a no-mirror control condition. Participants were exposed to one of the four mirror conditions depending on the day they signed up for the study. The primary independent variables were type of mirror condition, sex, and BMI, and the primary dependent variable was the dichotomous behavior of stair or elevator use.

### Materials and Measures

#### Mirror Manipulation

The standard mirror condition exposed participants to a regular, full-length framed mirror that shows the entire body, in order to induce public self-awareness (20). The thinning mirror and widening mirror conditions used the same mirror as the standard condition, but the mirror was bent to be concave in the middle to make participants’ reflections appear slightly thinner and bent to be convex in the middle to make participants’ reflections appear slightly wider than they would normally be. The participants assigned to the control condition were not exposed to a mirror. To determine whether or not participants actually saw themselves in the mirror, the item, “Did you see your reflection in a mirror near the stairs and elevator on the first floor of the parking garage?” appeared at the end of the questionnaire. The participants who answered “yes” to this question were then prompted to answer how their reflection appeared to them using the response options, “it appeared as it normally does,” “it appeared wider than normal,” and “it appeared thinner than normal.”

#### Stair and Elevator Use

Participants’ stair or elevator use was assessed through investigator observation. Members of the research team recorded the method of transportation used by each participant along with their condition (i.e., standard mirror, thinning mirror, widening mirror, or no-mirror control).

#### Demographics

A questionnaire administered online *via* the software system Qualtrics was used to collect participants’ demographic information, including their age, sex, race, ethnicity, height, body weight, and physical injury/disability status. Questions assessing participants’ sex, race, ethnicity, and physical injury/disability status were modeled on Dorsey and Graham (34), who include ethnicity as a separate measure from race to assess participants’ specific Hispanic, Latin, or Spanish background (e.g., Mexican, Cuban, etc.). Height and body weight were used to calculate each participant’s BMI in kilograms per square meter. BMI calculations were also used to create a “weight status” variable in which participants were labeled according to the body weight categories outlined by the World Health Organization (35) as either underweight (BMI < 18.5 kg/m^2^), normal weight (BMI = 18.5–24.9 kg/m^2^), overweight (BMI = 25–29.9 kg/m^2^), or obese (BMI ≥ 30 kg/m^2^).

#### Active Transportation, Physical Activity, and Weight-Control Behaviors

Participants’ primary method of campus transportation was assessed with the item, “What is your primary method of transportation on and around campus?” Response choices included more active methods (e.g., walking) and more inactive methods (e.g., motor vehicle) of transportation. Participants’ frequency of strenuous, moderate, and mild PA was then measured with three items from the Project Eat-III Survey for young adults (36). The items were modified from Godin and Shepard (37) and Sallis et al. (38). Finally, weight-control behaviors were assessed using three different items from the Project Eat-III Survey for young adults (39, 40). These items assessed participants’ use of unhealthy (e.g., fasting) and healthy (e.g., eating more fruits and vegetables) strategies for weight control.

#### Self-Awareness

To assess participants’ self-awareness, the situational self-awareness scale (SSAS) developed and empirically tested by Govern and Marsch (20) was administered. The 9-item scale asked participants to rate the extent to which they agree (1 “strongly disagree” to 7 “strongly agree”) with statements about their public and private awareness, as well as their awareness of immediate surroundings based on how they were feeling right at that moment (20). Sample items from each category of awareness include “right now, I am concerned about the way I present myself” (public), “right now, I am conscious of my inner feelings” (private), and “right now, I am keenly aware of everything in my environment” (surroundings). The SSAS has been determined to be a valid and reliable scale for assessing self-awareness and distinguishing between public, private, and environmental awareness (20).

#### Body Size Perception

To measure participants’ perceived body size and determine whether exposure to a distorted mirror influenced this perception, the Contour Drawing Rating Scale [CDRS; (41)] was used. The CDRS includes nine female and nine male front-view contour drawings that sequentially increase in body size from left to right (Drawing 1 being the thinnest and Drawing 9 being the largest body size). Participants were asked to identify the sex-matched drawing that most closely resembles their own body. The CDRS has been determined to be a valid and reliable (*r* = 0.78, *p* < 0.001) scale for measuring body-size perception (41).

### Procedure

Participants selected an available date to arrive individually at a campus parking garage and were exposed to one of the four conditions previously described. Mirrors for the three mirror conditions were placed on an easily viewed wall approximately equidistant from the stairs and the elevator in the garage. The no-mirror condition did not have a mirror on the wall. Participants were instructed to meet near the Parking Services office on the first floor of the enclosed parking garage. Signs were posted directing participants to a check-in table inside placed in front of the wall with the mirror. On the check-in table, there were instructions telling participants to go to the fourth floor at their study time where a researcher would administer the questionnaire. The researcher on the fourth floor of the garage privately recorded the participant’s condition and transportation method (stairs or elevator) and then greeted the arriving participant. Participants provided informed consent and then completed an online questionnaire from a laptop that confidentially assessed their demographic information, preferred method of transportation around campus, PA behaviors, weight-control behaviors, self-awareness, body size perception, and the appearance of a mirror and their reflection. All participants were thanked and debriefed as to the true nature of the study following completion of the questionnaire.

### Statistical Analysis

To test for differences in conditions among key variables, chi-square analyses (for the categorical variables of sex and BMI category) and *t*-tests and analyses of variance (ANOVA; for the continuous variables of body size perception and self-awareness) were performed.

Logistic regression models were run in SPSS 17.0 to test the primary study hypotheses, controlling for covariates taken from past research: age, sex, race (white versus non-white), ethnicity [not Hispanic, Latino(a), or of Spanish origin versus Hispanic, Latino(a), or of Spanish origin], BMI, preferred method of transportation around campus (dichotomized into active versus non-active), PA behaviors (total mild, moderate, and strenuous), weight-loss behaviors (total healthy versus unhealthy), self-awareness (total private, public, and immediate surroundings), and body size perception. To test Hypothesis 1, that individuals exposed to mirrors would show more stair use than those not exposed to a mirror, all three mirror conditions were combined into one “mirror” condition; then predicted odds of stair use were compared between the mirror and no-mirror groups. Hypothesis 2, that males would show greater stair use than females, was tested by comparing predicted odds of stair use among males and females. To test Hypothesis 3, that BMI would be inversely related to stair use, the BMI variable was used to predict stair use. Finally, Hypothesis 4, that distorting mirrors would impact stair use, was tested by comparing stair use among those in the thinning and widening mirror conditions. For Hypothesis 4, logistic regression examined mirror conditions separately with a dummy-coded variable using standard mirror as the reference group.

## Results

After excluding the data of participants who opted out after learning the study had used deception to manipulate mirrors and/or who reported having a physical injury that makes it difficult to walk or climb stairs (*N* = 7), a total of 167 students who took either the stairs or the elevator to the fourth floor of the parking garage study location remained. A majority (51%) of the sample identified as males, 18–25 years old (96%), White (88%), and not Hispanic, Latino/a, or of Spanish ethnic descent (87%; see Table 1). Mean BMI for the sample was 22.6 kg/m^2^; most participants had a BMI within the normal range (70.9%), while 18.2% were within the overweight BMI range and 5.5% fell into each of the underweight and obese BMI categories. Chi-square analyses revealed that participants’ sex, χ^2^(3) = 1.93, *p* = 0.59, and BMI category, χ^2^(9) = 7.09, *p* = 0.63, did not significantly differ by experimental condition. Additionally, a one-way ANOVA indicated that participants’ body size perception did not significantly differ across the four conditions, *F*(3, 163) = 0.28, *p* = 0.84.

**Table 1:** **Descriptive statistics for stair use covariates (*N* = 167)**.

Covariates	No mirror	Standard mirror	Thin mirror	Wide mirror	Total
*n*	38	45	45	39	167
Male	17 (45.9%)	24 (54.5%)	27 (61.4%)	21 (42.9%)	89 (51.1%)
White	31 (91.2%)	34 (77.2%)	41 (93.2%)	35 (89.7%)	141 (87.6%)
Non-Hispanic	31 (83.8%)	38 (88.4%)	38 (86.4%)	32 (88.9%)	139 (86.9%)
BMI	23.1 (3.9)	22.1 (2.8)	22.4 (4.0)	22.7 (3.6)	22.6 (3.6)
Body perception – out of 9	5.3 (1.3)	5.1 (1.3)	5.3 (1.2)	5.2 (1.3)	5.2 (1.3)
Active transportation	24 (64.9%)	30 (66.7%)	26 (60.5%)	32 (82.1%)	112 (68.3%)
SA – out of 45	31.9 (4.1)	31.7 (4.8)	32.0 (3.8)	32.8 (5.6)	32.1 (4.6)
Total exercise – out of 24	12.2 (6.3)	10.7 (5.1)	13.2 (6.7)	13.7 (6.9)	12.4 (6.3)
Unhealthy WC – out of 9	0.8 (1.4)	0.8 (1.3)	0.9 (1.5)	1.5 (1.9)	1.0 (1.6)
Healthy WC – out of 12	6.3 (2.9)	5.3 (3.2)	6.1 (3.2)	5.7 (3.5)	5.8 (3.2)

*For categorical covariates (male, White, non-Hispanic, and active transportation), label reflects reference group. BMI, body perception, SA, total exercise, unhealthy WC, and healthy WC are continuous variables*.

*BMI, body mass index; SA, self-awareness; WC, weight-control behaviors*.

### Self-Awareness

The mean self-awareness (SA) score for the sample was 32.1 (out of 45) and 9.0 (out of 15) for public self-awareness, which is comparable to mean SA scores found elsewhere in collegiate samples (20). SA means and SDs for the four experimental conditions are shown in Table 1. Results from a one-way ANOVA indicated that participants’ mirror condition was not significantly associated with their overall self-awareness, *F*(3, 157) = 0.42, *p* = 0.74 or their public self-awareness, *F*(3, 159) = 0.56, *p* = 0.65. In addition, an independent-samples *t*-test was conducted to assess if overall self-awareness and public self-awareness differed as a function of whether participants reported seeing a mirror [*N* = 83 (49.7%)] or not [*N* = 84 (51.3%)] near the stairs and elevator. Results indicated that neither type of self-awareness was affected by consciously seeing a mirror [overall SA: *t*(155) = 0.20, *p* = 0.84; public SA: *t*(157) = 0.83, *p* = 0.41].

### Collinearity of Predictor Variables

To assess the relationships between key covariates identified from previous research, a correlation matrix was run along with the base logistic regression model. No strong correlations between any of the variables were indicated, so all variables were retained in the subsequent models.

### Hypotheses Testing

Overall, more participants took the stairs (61.7%) than the elevator (38.3%). For all four hypotheses, logistic regression was performed to determine the odds of stair (versus elevator) use controlling for 10 covariates identified from previous research: sex, race, ethnicity, BMI, body size perception, use of active transportation, overall self-awareness, exercise behavior, unhealthy weight-control behaviors, and healthy weight-control behaviors. Age was excluded as a covariate due to the homogeneity of the sample (all participants were enrolled in an introductory psychology course and 96.3% identified as being within the age of 18–25 years). For Hypothesis 1, the odds of stair use were compared among participants in the no-mirror control condition and participants in any of the three mirror conditions. Overall, this model predicted approximately 28% of the variance in stair use and correctly classified 72.3% of cases [χ^2^(11) = 32.71, *p* = 0.001]. Participants’ mirror exposure, sex, BMI, total exercise, and both unhealthy and healthy weight-control behaviors significantly predicted their odds of stair use (see Table 2). Surprisingly, significantly more participants who were exposed to a mirror (39.5%) took the elevator over the stairs than participants who were not exposed to a mirror (34.2%), holding all covariates constant [odds ratio (OR) = 0.37, *p* = 0.04]. The lower stair use among those in the mirror conditions thus failed to support Hypothesis 1.

**Table 2:** **Odds ratios (OR) for stair use covariates**.

Covariates	OR	*p*	95% CI
Mirror	0.37	0.04	0.14–0.96*
Female	0.33	0.02	0.13–0.85*
Non-White	0.47	0.22	0.14–1.56
Hispanic	0.71	0.59	0.20–2.50
BMI	0.84	0.05	0.71–0.99*
Body perception	1.15	0.54	0.74–1.80
Inactive transportation	0.62	0.25	0.28–1.39
SA	1.01	0.88	0.92–1.10
Total exercise	1.09	0.02	1.02–1.18*
Unhealthy WC	1.55	0.01	1.14–2.11*
Healthy WC	0.85	0.04	0.72–0.99*

*All analyses presented are adjusted for covariates. For ease of interpreting categorical covariates (mirror, female, non-White, Hispanic, and inactive transportation), the label reflects the group associated with the direction of the OR. Therefore, an OR <1.0 indicates the covariate has an inverse relationship with (decrease in) stair use. BMI, body perception, SA, total exercise, unhealthy WC, and healthy WC are continuous variables*.

*BMI, body mass index; SA, self-awareness; WC, weight-control behaviors*.

*p < 0.05.

Hypothesis 2 was supported, as significantly more males (73.0%) than females (49.3%) chose to take the stairs (OR = 0.33, *p* = 0.02). The logistic regression results revealed that males were more likely to take the stairs than females even after controlling for race, ethnicity, BMI, body size perception, use of active transportation, overall self-awareness, exercise behavior, unhealthy weight-control behaviors, and healthy weight-control behaviors (see Table 2). Holding these same covariates constant, participants’ BMI was also significantly predictive of stair use (OR = 0.84, *p* = 0.049) such that higher BMI was associated with a reduction in likelihood of stair use, thus supporting Hypothesis 3. *Post hoc* analyses were conducted investigating whether there was an interaction between mirror condition and each of the sex and BMI variables, but no significant interaction was detected for either. Notable continuous predictors that were significantly positively associated with greater likelihood of stair use were overall exercise participation (OR = 1.09, *p* = 0.02) and participation in unhealthy weight-loss behaviors (OR = 1.55, *p* = 0.01; confidence intervals for these covariates are displayed in Table 2). Interestingly, participation in healthy weight-loss behaviors, such as exercising more and eating more fruits and vegetables, was inversely related to stair use such that as the number and frequency of healthy weight-loss behaviors increased, the likelihood of stair use decreased (OR = 0.85, *p* = 0.04).

To address Hypothesis 4 by exploring how exposure to a thinning mirror or a widening mirror influences stair use, a second logistic regression model was run that compared each of the thinning and widening mirror conditions to the standard (non-distorted) mirror condition, which served as the reference group. Overall, this model significantly predicted stair use, χ^2^(13) = 33.41, *p* = 0.001, but both mirror-type predictors were non-significant (thinning mirror: OR = 0.68, *p* = 0.49; widening mirror: OR = 0.64, *p* = 0.46) (see Table 3). Regarding the exploratory question posed by Hypothesis 4, these results indicate that neither thinning nor widening mirrors were associated with increased stair use, and these mirrors may even trend toward the opposite direction of decreasing stair use.

**Table 3:** **Odds ratios (OR) for stair use among individual mirror conditions**.

Covariates	OR	*p*	95% CI
No mirror	2.08	0.21	0.66–6.55
Thin mirror	0.68	0.49	0.23–2.01
Wide mirror	0.64	0.46	0.20–2.06
Female	0.33	0.02	0.13–0.86*
Non-White	0.44	0.19	0.13–1.49
Hispanic	0.71	0.60	0.20–2.53
BMI	0.84	0.05	0.71–1.00
Body perception	1.16	0.51	0.74–1.82
Inactive transportation	0.61	0.23	0.27–1.37
SA	1.01	0.80	0.93–1.11
Total exercise	1.10	0.02	1.02–1.18*
Unhealthy WC	1.57	0.01	1.15–2.14*
Healthy WC	0.85	0.05	0.72–1.00

*All analyses presented are adjusted for covariates. Categorical covariate label reflects the group associated with the direction of the OR. An OR <1.0 indicates the covariate has an inverse relationship with stair use. BMI, body perception, SA, total exercise, unhealthy WC, and healthy WC are continuous variables*.

*OR, odds ratio; BMI, body mass index; SA, self-awareness; WC, weight-control behaviors*.

*p < 0.05.

## Discussion

This study examined predictors of a person’s choice to take the stairs or the elevator to a higher floor, specifically focusing on how the presence of a mirror and the individual’s perceived body size influence this choice. Contrary to what was hypothesized, it was found that more people who were exposed to one of three mirror conditions chose to take the elevator over the stairs than those who were not exposed to a mirror. Although previous research has indicated that full-length mirrors that show the entire body can heighten overall self-awareness and induce public self-awareness particularly (20), self-awareness was not found to significantly differ across the mirror and the no-mirror control conditions in the present study. Therefore, it is plausible to assume that the hypothesis of increased stair use among the mirror conditions was not supported because participants’ self-awareness was not affected and thus they did not mentally compare themselves to an ideal standard, as postulated by OSA theory (21).

In addition, many participants could have high extrinsic motivation for engaging in PA, as has been found to be common among college students (42). Individuals with greater extrinsic than intrinsic motivation for certain activities, including PA, tend to engage in these activities for reasons focused on weight and appearance more than reasons such as challenge or personal enjoyment (42). In this study, the college students who were exposed to a mirror may have chosen the physically inactive alternative (i.e., elevator use) if they did not believe that selecting the physically active option (i.e., stair use) would positively impact their recently seen appearance (e.g., by losing weight).

Furthermore, feelings of body dissatisfaction or being far from an ideal PA standard could have impacted students’ decision to use the elevator over the stairs. Research by Martin Ginis et al. (43) supports the idea that body or PA dissatisfaction could deter stair use with the discovery that inactive people who exercised in front of a mirror reported more negative emotions associated with the exercise experience than those who exercised in front of a non-mirrored wall. Consequently, it could be argued that individuals who are not regularly active and who are reminded of this after seeing themselves in a mirror have fewer positive reasons and less desire to be active by using the stairs. Indeed, this study’s finding that less total exercise participation is associated with less stair use also supports such conjecture.

In addition to differences in stair use after exposure to a mirror, the present study found sex differences in the choice to use the stairs or the elevator. Overall, more males used the stairs than females, which is consistent with past research on both stair use (15, 29) and general exercise participation (3). Although males were more likely to use the stairs overall, no significant sex-by-mirror exposure interaction was found. This indicates that the presence of a mirror did not significantly alter the substantial already-existing differences in stair use based on sex. Along with participant sex, BMI was predictive of stair use such that those with lower BMI were more likely to use the stairs than those with higher BMI, a finding also supported by previous research (29). Although Proffitt’s (30) economy of action theory explains that individuals with lower BMI may be more likely to use the stairs due to their perception that less energy is required than those with higher BMI and college women typically have lower BMI values than men (44), other research suggests that women are significantly more likely to have an inflated body weight perception than men (32). It may have been the women’s perception of rather than their actual body weight that deterred stair use in this study, as an inflated weight perception may have led to a belief that stair climbing would be more effortful. The idea that an inflated body weight perception could have led women to avoid using the stairs needs to be explored further, however, because Sugovic and colleagues (31) recently found that only actual body weight, not beliefs about weight, impacted distance judgments for both males and females. Women also tend to have greater body weight concerns than men (42), making it again plausible that general feelings of body dissatisfaction, with or without mirror exposure, also help explain why fewer females than males took the stairs in the present study.

When compared to the standard, non-distorted mirror condition and controlling for all other covariates, neither participants in the thinning or widening mirror condition showed significantly increased odds of taking the elevator over the stairs. Furthermore, the odds of elevator use in the two distorted mirror conditions were similar, implying that there were no differences in behavior based on whether a person’s body size appeared slightly thinner or wider than normal. The fact that body size perception did not significantly differ across mirror conditions in this study supports the postulation that distortions to the thinning and widening mirror were subtle and may not have appeared different enough to consciously impact participants’ perception of their body size.

Another interesting finding from the present study that should be noted is the fact that unhealthy weight-loss behaviors were positively associated with stair use, whereas healthy weight-loss behaviors were negatively associated with stair use. That is, individuals who engaged in behaviors such as skipping meals and taking diet pills showed increased odds of taking the stairs, whereas individuals who more frequently engaged in behaviors such as exercise and watching portion sizes showed lower odds of taking the stairs. Although exercise was included as a “healthy” weight-control behavior and more total exercise participation was associated with greater likelihood of stair use, it could be that participants indicating their engagement in exercise as part of their overall healthy weight-loss behavior are more extrinsically motivated to perform PA than those with higher overall exercise participation because they are focusing more on controlling their weight (appearance) in the first place. So, perhaps type of motivation is explaining these seemingly contradicting findings regarding participants’ exercise behavior, such that those who exercise more overall take the stairs more because they enjoy being active or desire to improve their health, whereas those who exercise as part of a weight-control strategy take the stairs less because they do not view stair use as an opportunity to improve their appearance. Previous research supports such a notion, as appearance and weight-related motives for exercise versus health and fitness-related motives, have been found to be inversely associated with exercise participation (45).

There are limitations to the present study that warrant discussion. First, several participants who were in a mirror condition reported not seeing a mirror (about 25% of this portion of the sample). It could be problematic if these individuals truly did not see a mirror when one was present because its influence on self-awareness and stair use would be nullified. Because the mirror was large and placed at eye-level near the participant instructions sheet, it is possible that participants unconsciously registered the presence of a mirror, but later did not consciously recall having seen the mirror. However, if most of these participants really failed to notice the mirror, it would behoove future research to assess how self-awareness might influence behavior with a more distinctive mirror, such as one that is an odd shape or has been painted a bright color. Second, although this study purposely had participants arrive one at a time to avoid social influence on transportation choice, some participants’ behaviors could have been impacted by the presence of other people in the parking garage. It is assumed that the issue of social influence was minimal in the present study, though, because the part of the parking garage where the questionnaire was administered typically has low pedestrian traffic, particularly to the upper floors. Finally, the diversity of the study sample was limited; future research should attempt to investigate stair use among older students, minorities, and those outside of the college environment, such as adults working in multilevel office buildings.

Several important implications also arose from the present study and are worth consideration. Even though the current results reveal that exposure to mirrors may actually decrease stair use among college students, other research has indicated that this population is likely to have extrinsic (e.g., weight, appearance) rather than intrinsic (e.g., enjoyment, health) motives for performing PA. Furthermore, extrinsic motives are likely the result of enhanced public self-awareness because public self-awareness implies the possibility of social evaluation (28, 46). Taken together, these findings imply that future mirror interventions may be more successful at increasing PA among college students if the students are exposed to both a mirror and an appealing reason to engage in the behavior. For example, if college students are primarily motivated to engage in PA by external sources such as other people, normative signage could be placed near the mirror that encourages stair use by stating that most other students take the stairs. Results from this study are consistent with such a message: nearly 62% of the present sample chose the stairs over the elevator. Moreover, recent research has demonstrated that exposure to descriptive norm signs stating that most people use the stairs led to a significant decrease in elevator use on a college campus (47).

In sum, the present study assessed stair versus elevator use on a college campus by students who were either exposed to a full-length mirror or to no-mirror. Students who were not exposed to a mirror, males, those with lower BMI, those with higher total exercise participation, and those who engaged in more unhealthy weight-control behaviors demonstrated significantly increased odds of taking the stairs. Conversely, students who were exposed to a mirror showed increased odds of taking the elevator. Although not measured in this study, high appearance-focused motivation for engaging in PA among college students may be an explanation for the mirrors’ effect on stair use and should be examined further. Nevertheless, a majority of students chose the stairs over the elevator and thus descriptive norm interventions, used in conjunction with mirror exposure, may result in higher rates of stair use. The health benefits of stair climbing are noteworthy, and findings from the present study provide an important stepping stone for future research aiming to promote stair use over more inactive floor-climbing methods, such as elevator or escalator use.

## Author Contributions

KH (first author) collected the data, conducted the statistical analyses, wrote the first draft of the manuscript, and revised the manuscript. DG conceived the study idea, oversaw study design, data collection, analysis, and writing, and performed critical revision of the manuscript. Both authors have approved the submitted version of the manuscript.

## Conflict of Interest Statement

The authors declare that the research was conducted in the absence of any commercial or financial relationships that could be construed as a potential conflict of interest.
